# GrapeSLAM: UAV-based monocular visual dataset for SLAM, SfM and 3D reconstruction with trajectories under challenging illumination conditions

**DOI:** 10.1016/j.dib.2025.111495

**Published:** 2025-03-18

**Authors:** Kaiwen Wang, Sergio Vélez, Lammert Kooistra, Wensheng Wang, João Valente

**Affiliations:** a Information Technology Group, Wageningen University & Research, Wageningen, 6708 PB, the Netherlands; b Group Agrivoltaics, Fraunhofer Institute for Solar Energy Systems ISE, Freiburg, 79110, Germany; c Laboratory of Geo-Information Science and Remote Sensing, Wageningen University & Research, Wageningen, 6708 PB, the Netherlands; d Agricultural Information Institute, Chinese Academy of Agriculture Sciences, Beijing, 10086, China; e Centre for Automation and Robotics (CAR), Spanish National Research Council (CSIC), Madrid, 28500, Spain

**Keywords:** Visual SLAM, Precision agriculture, UAV, RTK GNSS data, Woody crop

## Abstract

SLAM (Simultaneous Localization and Mapping) is an efficient method for robot to percept surrendings and make decisions, especially for robots in agricultural scenarios. Perception and path planning in an automatic way is crucial for precision agriculture. However, there are limited public datasets to implement and develop robotic algorithms for agricultural environments. Therefore, we collected dataset “GrapeSLAM”. The ``GrapeSLAM'' dataset comprises video data collected from vineyards to support agricultural robotics research. Data collection involved two primary methods: (1) unmanned aerial vehicle (UAV) for capturing videos under different illumination conditions, and (2) trajectories of the UAV during each flight collected by RTK and IMU. The UAV used was Phantom 4 RTK, equipped with a high resolution camera, flying at around 1 to 3 meters above ground level.

Specifications Table

**Table:** 

Subject	Agricultural Sciences, Agronomy, Robotics and Crop Science
Specific subject area	Precision Agriculture for agricultural automation with UAVs.
Type of data	“.MOV”: raw video data collected by high resolution camera of UAV “.xlsx” datasheet: description of the UAV flight status
Data collection	Unmanned Aerial Vehicle: - DJI Phantom4 RTK (integrated sensor). Sensor characteristics: - Focal aperture range: f2.8 - f.11 - Shutter speed: 8-1/8000 s Flight details: - Flight speed: not stable, between 0.1 and 1 m/s. - Flight altitude: around 1 to 3 m above ground level (AGL) A total of 25 videos and their trajectories are provided. The trajectories contains many status of UAV including pitch, roll, compass, speed in x, y, z-asix, and etc. The names of the trajectories are same as the name of videos, such as DJI0016.xlsx and DJI0016.MOV. Each flight are manually controlled by drone pilot within vineyard rows with different camera view directions under challenging illumination conditions.
Data source location	Institution: Wageningen University & Research City/Town/Region: Tomiño, Pontevedra, Galicia Country: Spain Latitude and longitude (and GPS coordinates) for collected samples/data: 41°57′18.5″N 8°47′41.2″W
Data accessibility	Repository name: Zenodo Data identification number: 10.5281/zenodo.14658375 Direct URL to data: https://zenodo.org/records/14658376
Related research article	Wang, K., Kooistra, L., Wang, Y., Vélez, S., Wang, W., & Valente, J. (2024). Benchmarking of monocular camera UAV-based localization and mapping methods in vineyards. *Computers and Electronics in Agriculture, 227*, 109661. https://doi.org/10.1016/j.compag.2024.109661

## 1 Value of the Data

• Public datasets with UAV's trajectories are helpful for researchers to perform and develop SLAM and path planning algorithms in agricultural environments..
• RTK GNSS data provides high accurate position information of the UAV.
• Datasets with multiple-perspective videos and challenging illumination conditions are crucial for precision agriculture.
• The dataset promotes cross-disciplinary studies in viticulture, remote sensing, robotics, and computer vision, enhancing innovation and collaboration. It enables new methods for agriculture automation, and precision agriculture, advancing research techniques.
• This dataset can be used for SLAM and also for simulation and synthetic data generation, agricultural robots test, 3D reconstruction, digital twin in agriculture.
• This dataset can be integrated with other datasets [[1], [2], [3], [4]] from the same vineyard that contain multiple information such as the position of the plant trunks, LiDAR point cloud, frame annotations, and multispectural images, to leverage understanding of the vineyard environment for robots.

## 2 Background

Currently, labour shortages and environmentally harmful practices impact traditional labour-intensive agricultural production. To address the issue, precision agriculture with state-of-the-art computer vision and robotic technologies should apply to the agricultural domain [5]. However, due to the complex, unstructured, and dynamic features in agricultural environments, it is difficult to collect and organize a standard dataset for benchmarking algorithms [6]. While UAV datasets like EuRoC [7] provide stereo camera images, IMU, and ground truth 6D poses for SLAM in controlled indoor environments,agriculture requires datasets that reflect the challenges of outdoor settings. These include variable lighting, dense vegetation, and the need for accurate positional data from diverse perspectives. Existing agricultural datasets often focus on specific data types, such as LiDAR or multispectral imagery, but rarely integrate visual data with precise trajectory information.

The GrapeSLAM dataset fills this gap by offering UAV videos with a monocular camera, IMU, and RTK-GNSS position of UAV from a real-world vineyard. These videos included a variety of flight scenarios and were collected under different conditions, including variable illumination conditions, different camera perspectives, and flights with and without loop closure. Moreover, this dataset can be synergistically combined with other existing datasets from the same vineyard [[1], [2], [3], [4]], which encompass a diverse range of data types, including videos, UAV orthoimages, LiDAR, and MOTS annotations of grapes, trunks and poles. This diversity enhances the potential for robotics, computer vision, and 3D reconstruction research, allowing for developing and evaluating semantic or object based SLAM, automatic path planning, and large scale 3D reconstruction algorithms. SLAM is a key technology for autonomous robots to localize and map surrounding without any prior knowledges. SLAM, combined with path planning and 3D reconstruction, can enable robots with higher prerception and control intelligence.

Therefore, GrapeSLAM aligns with the goals of precision agriculture by supporting efforts to improve efficiency and automation in farming, creating a rich multimodal foundation for advancing robotic perception and decision-making in viticulture. In order to provide a diverse and standard public dataset for UAV-based visual SLAM in the agricultural domain, the dataset is essential for the field robotic community.

## 3 Data Description

The dataset was collected during the 2023 harvesting campaign between September 18th and 19th in a 1.06-hectare commercial vineyard (*Vitis vinifera* cv. Loureiro) located in Tomiño, Spain (41°57′18.3″N 8°47′41.9″W). The plants, managed in a vertical trellis system, were planted in 1990 with an NE-SW orientation. The distance between rows and plants is 3 × 2.5 meters, respectively, and no leaf removal was performed, resulting in a dataset marked by leaf occlusion.

The videos in the dataset was collected by flying the UAV within the vineyard rows manually. Three flight modes were conducted: [1] the camera perspective is side view to the vineyard row, [2] the camera perspective is front view to the path between two rows, and [3] the camera perspective is side view to the vineyard row with a loop closure.

**Fig 1:**
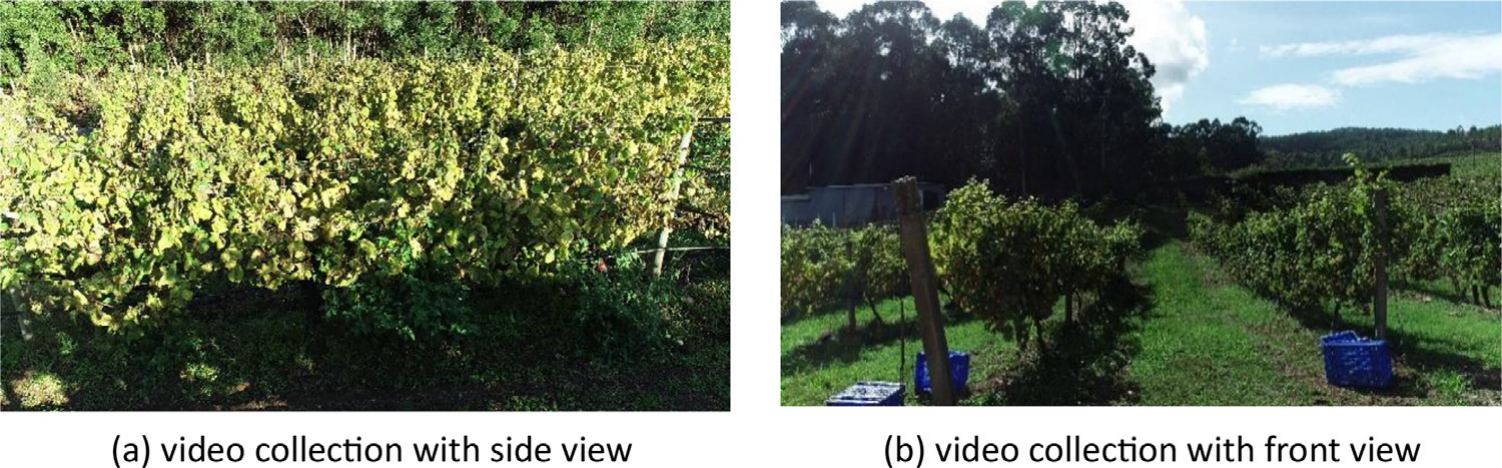
Flight modes during video collection. (a) shows the camera perspective with side view, (b) shows the camera perspective with front view.

The dataset consists of 24 high-resolution video files (.MOV) with different illumination conditions and flight modes (Fig. 1). The UAV flight modes The videos and trajectories share identical filenames for straightforward pairing (e.g., DJI0016.MOV and DJI0016.xlsx) (Table 1).

**Table 1:** Description of the datasets.

Video name	Perspective	Video Duration	Average flight speed (m/s)	Average flight height (m)	Illumination conditions	Loop Closure
DJI0016.MOV	Front	54 s	0.98	1.82	Half sunlight, half shadow	No
DJI0018.MOV	Side	55 s	0.92	3.20	Half sunlight, half shadow	No
DJI0019.MOV	Side	1 min 11 s	0.70	2.70	Most sunlight, little shadow	No
DJI0027.MOV	Front	1 min 16 s	0.65	1.58	Half sunlight, half shadow	No
DJI0028.MOV	Side	1 min 08 s	0.73	2.50	Most shadow, part sunlight	No
DJI0029.MOV	Side	1 min 17 s	0.62	2.87	Half sunlight, half shadow	No
DJI0032.MOV	Front	1 min 05 s	0.77	1.70	Half shadow, half sunlight	No
DJI0033.MOV	Side	1 min 30 s	0.53	2.60	Part sunlight, part shadow	No
DJI0034.MOV	Side	1 min 22 s	0.60	2.51	Most shadow, part sunlight	No
DJI0037.MOV	Front	1 min 25 s	0.58	1.70	Most sunlight, part shadow	No
DJI0038.MOV	Side	1 min 20 s	0.61	2.40	Most sunlight, part shadow	No
DJI0042.MOV	Side	1 min 28 s	0.55	2.95	Most sunlight	No
DJI0044.MOV	Front	1 min 27 s	0.54	1.76	Half shadow, half sunlight	No
DJI0046.MOV	Side	1 min 57 s	0.43	2.83	Half sunlight, half shadown	No
DJI0047.MOV	Side	1 min 28 s	0.58	2.73	Part shadow, most sunlight	No
DJI0048.MOV	Side	1 min 31 s	0.55	2.69	Most sunlight, part shadow	No
DJI0049.MOV	Front	1 min 37 s	0.48	1.79	Half shadow, half sunlight	No
DJI0051.MOV	Side	1 min 42 s	0.48	2.53	Most sunlight, part shadow	No
DJI0054.MOV	Front	4 min 02 s	0.49	1.85	Part shadow, most sunlight	No
DJI0056.MOV	Side	7 min 20 s	0.58	2.24	Part shadown, most sunlight	Yes
DJI0058.MOV	Side	3 min 52 s	0.62	2.15	Most sunlight, part shadow	No
DJI0059.MOV	Side	3 min 51 s	0.55	2.35	Part shadow, most sunlight	No
DJI0060.MOV	Front	2 min 55 s	0.73	1.99	Most sunlight, part shadow	No
DJI0061.MOV	Side	6 min 36 s	0.69	2.25	Most sunlight, part shadow	Yes

The trajectories with RTK and IMU were collected at the same time during the flights. The state of the UAV is recorded every 100 ms.

## 4 Experimental Design, Materials and Methods

The UAV platform used in this study was the DJI Phantom4 RTK (DJI Sciences and Technologies Ltd., Shenzhen, Guangdong, China), equipped with an high resolution monocular RGB sensor, RTK-GNSS, and an inertial measurement unit (IMU).

**Fig. 2:**
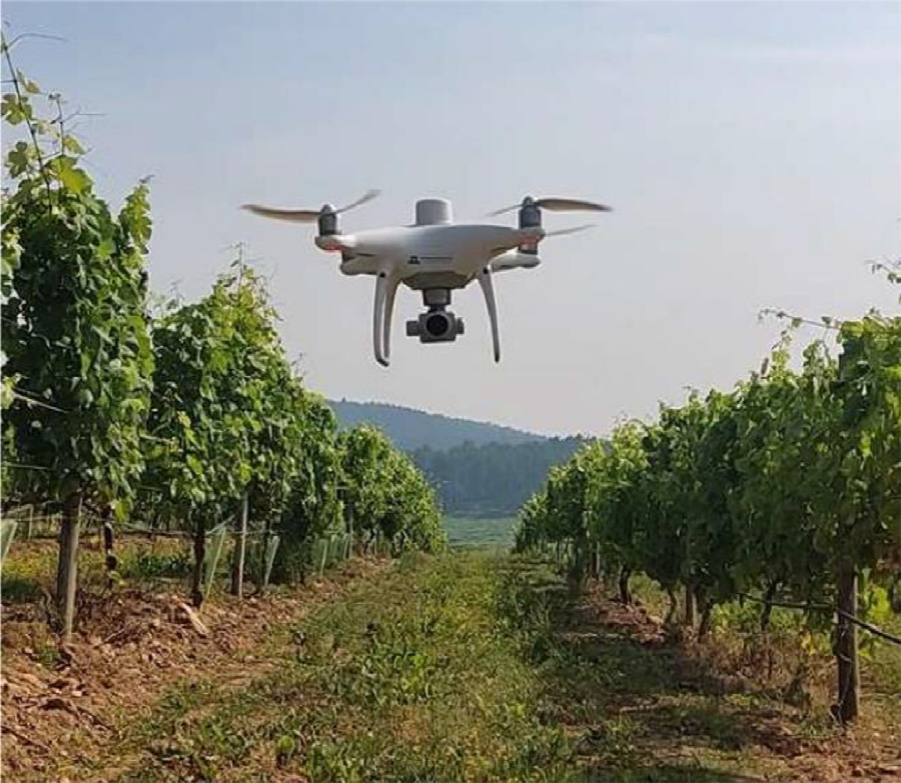
The position and pose of the UAV during flights (adapted from [4]).

The flights were conducted under a clear sky at 3 m AGL above the vineyard rows, with wind below 0.5 m/s, from three distinct flight perspectives: a side view parallel to vineyard rows, a front view along pathways between rows, and side views with loop closures. The UAV's trajectory data were logged every 100 ms, capturing flight parameters such as pitch, roll, compass orientation, and velocity. The flights were manually operated at speeds between 0.1 and 1 m/s (Fig. 2).

The trajectory data were recorded and generated from DJIFlightRecord log files with Airdata.^1^ And each trajectory file was aligned with video data based on their timestamp. To synchronise the video data and trajectories, the FPS of videos were reduced from 30 FPS to 10 FPS, which can match the trajectories timestamp. Thus, video and trajectory files were synchronized and checked for consistency to ensure accurate alignment between visual and positional data. The potential error sources could include IMU and RTK-GNSS error during data collection, which are inevitable.

## Limitations

Although the GrapeSLAM dataset provide a potential solution for agricultural robotic research, it has several limitations:

- The UAV's position and pose accuracy depend on the RTK and IMU systems in the DJI Phantom4 RTK. While RTK provides high positional accuracy, it can still be affected by factors like multipath errors, atmospheric conditions, and GNSS signal availability in the vineyard. IMU data may also experience drift over time, which can affect trajectory reconstruction.
- Data collection was conducted under ideal conditions, such as clear skies and low wind speeds (<0.5 m/s). These conditions don't fully represent challenges like wind or rain, which may limit the dataset's applicability in harsher environments.
- Since UAV flights were manually operated, differences in speed, altitude, and camera angles may introduce inconsistencies. While this reflects real-world variability, it could complicate the benchmarking of algorithms that require standardized inputs.
- Camera and Sensor Limitations: The dataset uses a high-resolution monocular RGB camera, but it lacks stereo vision or depth sensing. This may limit its usefulness for algorithms requiring depth data, such as 3D reconstruction or obstacle avoidance.
- Scalability to Other Vineyards: The dataset is from a single vineyard with a specific setup (vertical trellis system). Algorithms developed with this data may not generalize well to vineyards with different layouts, plant spacings, or canopy structures.

## Ethics Statement

The present work meets the ethical requirements for publication in Data in Brief. The work does not involve human subjects, animal experiments, or any data collected from social media platforms.

## Declaration of Generative AI and AI-Assisted Technologies in the Writing Process

During the preparation of this work, the authors used ChatGPT in order to improve the English language and avoid orthographic errors. After using this tool/service, the authors reviewed and edited the content as needed and take full responsibility for the content of the publication.

## CRediT authorship contribution statement

**Kaiwen Wang:** Visualization, Methodology, Data curation, Writing – original draft. **Sergio Vélez:** Investigation, Visualization, Methodology, Data curation, Writing – review & editing. **Lammert Kooistra:** Conceptualization, Supervision, Project administration, Writing – review & editing. **Wensheng Wang:** Conceptualization, Supervision, Project administration. **João Valente:** Conceptualization, Supervision, Project administration, Writing – review & editing.

## Data Availability

zenodoGrapeSLAM: UAV-based monocular visual dataset for SLAM with flight trajectories (Original data).

## Footnotes

1 https://airdata.com/.
